# Differences in adrenocortical responses between urban and rural burrowing owls: poorly-known underlying mechanisms and their implications for conservation

**DOI:** 10.1093/conphys/coaa054

**Published:** 2020-07-06

**Authors:** Antonio Palma, Julio Blas, José L Tella, Sonia Cabezas, Tracy A Marchant, Martina Carrete

**Affiliations:** 1 Department of Conservation Biology, Estación Biológica de Doñana (EBD-CSIC), 41092 Seville, Spain; 2 Department of Biology, University of Saskatchewan, S7N 5E2 Saskatoon, Saskatchewan, Canada; 3 Departament of Physical, Chemical and Natural Systems, Universidad Pablo de Olavide (UPO), 41013 Seville, Spain

**Keywords:** CORT, corticosterone, HPA axis, stress response, urbanization

## Abstract

The hypothalamus–pituitary–adrenal/interrenal (HPA) axis of vertebrates integrates external information and orchestrates responses to cope with energy-demanding and stressful events through changes in circulating glucocorticoid levels. Urbanization exposes animals to a wide variety of ever-changing stimuli caused by human activities that may affect local wildlife populations. Here, we empirically tested the hypothesis that urban and rural owls (*Athene cunicularia*) show different adrenocortical responses to stress, with urban individuals showing a reduced HPA-axis response compared to rural counterparts to cope with the high levels of human disturbance typical of urban areas. We applied a standard capture-restraint protocol to measure baseline levels and stress-induced corticosterone (CORT) responses. Urban and rural owls showed similar circulating baseline CORT levels. However, maximum CORT levels were attained earlier and were of lower magnitude in urban compared to rural owls, which showed a more pronounced and long-lasting response. Variability in CORT responses was also greater in rural owls and contained the narrower variability displayed by urban ones. These results suggest that only individuals expressing low-HPA-axis responses can thrive in cities, a pattern potentially mediated by three alternative and non-exclusive hypotheses: phenotypic plasticity, natural selection and matching habitat choice. Due to their different conservation implications, we recommend further research to properly understand wildlife responses to humans in an increasingly urbanized world.

## Introduction

Urbanization induces a wide range of ecological changes (Vitousek *et al.*, 1997), often acting as a selective in natural populations (Alberti, 2015), changing biotic and abiotic environmental factors, and having important consequences for biodiversity conservation worldwide. However, not all animals respond equally to these ecological changes and, while urbanization has been linked to lower diversity and abundance of some species (McKinney, 2008; Aronson *et al.*, 2014), other species are able to live successfully and even flourish in urban environments (Ellegren and Sheldon, 2008).

A growing amount of evidence shows that behaviour often defines the initial response of individuals toward anthropogenic changes and provides clues about how species cope with urbanization (see reviews by Lowry *et al.*, 2013; Sol *et al.*, 2013). Behavioural adjustments aimed at coping with human disturbance may be important for individuals to become urban dwellers (Lowry *et al.*, 2013) since urbanization is associated with high levels of human presence and other activities that are unusual in rural and/or natural habitats. Indeed, behavioural traits often differ between urban and non-urban conspecifics, potentially as a consequence of behavioural flexibility (i.e. phenotypic plasticity), selection of individuals with particular behaviours (i.e. natural selection) or non-random sorting of individuals among habitats (i.e. matching habitat choice). Carrete and Tella (2010, 2011) have proposed that bird species able to colonize urban habitats are those with higher inter-individual variability in their fear of humans (estimated as the distance at which a given individual flees when approached by a human, hereafter flight initiation distance FID). This suggests that from a pool of behaviourally variable rural individuals, only the less fearful ones can colonize cities (Carrete and Tella, 2011). As FID is a repeatable and heritable trait, this non-random distribution of individuals would likely arise through natural selection or matching habitat choice, although some degree of plasticity cannot be completely ruled out (Carrete and Tella, 2010, 2013; Carrete *et al.*, 2016).

However, the successful use of urban habitats may ultimately depend on how individuals within populations respond to the challenges imposed by urban environments (Romero *et al.*, 2009; Bonier, 2012). The hypothalamic–pituitary–adrenal/interrenal (HPA) axis of vertebrates plays a key role integrating external information and orchestrating hormonal and behavioural responses (Blas, 2015). The HPA axis is typically activated in response to increased energy demands (i.e. allostatic load) from both predictable environmental changes (e.g. causing daily and seasonal variations in baseline glucocorticoids, GC, levels) and unpredictable and potentially noxious stimuli (thus triggering stress-related GC elevations). Most studies on urban endocrine ecology have been performed using plasma samples from birds, and the results regarding baseline and stress-induced corticosterone (CORT) levels have revealed no consistent patterns of variation between urban and rural conspecifics (Bonier, 2012). However, circulating CORT levels are subjected to rapid changes following exposure to stress, and plasma samples typically provide information about the short-term endocrine state of the individual. To obtain a longer-term, integrated assessment of individuals’ endocrine state, Rebolo-Ifrán *et al*. (2015) tested for differences between urban and rural burrowing owls (*Athene cunicularia*) using feather CORT levels. Since CORT is deposited from the bloodstream into the feather structure over several weeks (i.e. the period of feather growth), feather CORT levels provide a longer-term, retrospective measure of HPA axis activity integrating both baseline and stress-induced plasma CORT levels (Bortolotti *et al.*, 2008; Blas, 2015). Consistent with the hypothesis that individuals occupy breeding sites with different levels of human disturbance according to their tolerance to humans (Carrete and Tella, 2010), they found no significant differences in feather CORT among urban and rural individuals. Nonetheless, the similar levels of CORT deposited in feathers may result from multiple stimuli—not only human presence—perceived by individuals, and the intensity of these stimuli may also vary between urban and non-urban habitats (Bonier, 2012; Rebolo-Ifrán *et al.*, 2015). Therefore, complementary approaches performed on the same species are necessary to better assess potential differences in the HPA axis activity associated with urban life.

In this study, we compared the adrenocortical response of urban and rural (non-urban) burrowing owls using the standard protocol of capture and restraint with repeated blood sampling (‘stress series’; Wingfield *et al.*, 1982). Based on our previous knowledge of our study model, we predicted that (i) urban and rural birds will show similar baseline CORT levels, as birds occupy sites according to their tolerance to human disturbance (Carrete and Tella, 2010, 2011; Rebolo-Ifrán *et al.*, 2015); (ii) rural birds will show higher variability in the CORT levels in the bloodstream, encompassing the variability shown by urban ones, as expected from previous behavioural studies assessing variability in fear of humans (Carrete and Tella, 2010, 2011); and (iii) urban birds will show lower stress-induced CORT levels, as only individuals with particular physiological responses are able to live close to humans without experiencing chronic stress (Lowry *et al.*, 2013; Rebolo-Ifrán *et al.*, 2015). Our results supported all of these predictions, contributing to a better understanding of how wildlife copes with urbanization. Although our study does not allow us to properly identify the mechanisms involved, we discuss the three alternative and non-mutually exclusive hypotheses that can explain these results, namely phenotypic plasticity, natural selection and matching habitat choice, and discuss the conservation implications derived from each scenario.

## Methods

### Study system

The burrowing owl is a small-sized Strigiform distributed across American open landscapes, breeding in self-excavated burrows or those excavated by fossorial mammals. Breeding pairs are territorial and show diurnal activity, and are easily located in the surroundings of their nest (Carrete *et al.*, 2016). Individuals breed for the first time when they are <1 year of age, although some individuals delay breeding (authors’ unpublished data).

The study site encompasses ca. 5400 km^2^ of urban and rural areas around the city of Bahía Blanca (Argentina). Rural owls breed in natural grasslands, pastures and cereal crops, where human presence is sporadic and largely restricted to a few, mostly unpaved roads. Urban owls nest in private and public gardens in residential areas, unbuilt spaces among houses, street curbs and boulevards, living in continuous contact with humans and intense road traffic. Previous studies show that urban owl populations were founded by individuals from the neighbouring rural areas a few decades ago (ca. 40 years; Mueller *et al.*, 2018). Individuals thriving in urban areas have experienced lower predation pressure, which allowed them to reach higher breeding success and densities compared to their rural counterparts (Rodríguez-Martínez *et al.*, 2014; Rebolo-Ifrán *et al.*, 2017). For more details, see Carrete and Tella (2010, 2011, 2013), Rebolo-Ifrán *et al.* (2015, 2017) and Mueller *et al*. (2018, 2020).

### Field procedures

During the chick-rearing period (December to January), we performed a standardized-capture and restraint protocol (Wingfield *et al.*, 1982) in 27 breeding adults (urban: 7 females and 7 males; rural: 7 females and 6 males), which were captured using bownets at their nests during daylight. All captured individuals were attending pre-fledging chicks of similar ages (between 3 and 4 weeks, estimated visually by their size and plumage development), thus controlling for potential effects of breeding phenology on CORT (Blas, 2015). We serially obtained five blood samples (100 μL each) per individual from the brachial vein at pre-determined time intervals following capture (i.e. within the first ca. 3 min post-capture as well as 15, 30, 45 and 60 min post-capture). Birds were housed individually in dark metal cages (20 × 15 × 15 cm) between successive bleedings. Blood samples were preserved in a plastic storage box surrounded by ice coolers, inside an isothermal bag, until centrifuging (10 min at 10577 g) within the same day. Plasma was stored at −80°C until the determination of CORT levels (see below). After blood sampling, we visually assessed sex (Carrete and Tella, 2010; unpublished results) and weighed and measured (wing chord) each individual. The first blood sample obtained from each individual was collected within ca. 3 min after capture (mean = 2.52 min; SE = 0.69). CORT titres in this sample were unrelated to time post-capture (*F*_1,25_ = 0.16, *p* = 0.69) and were thus considered to be a good estimate of baseline CORT levels (Romero and Reed, 2005). Baseline CORT levels were also unrelated to the time lag between trap setting and bird capture (mean = 31.24 min; SE = 30.95, *F*_1,25_ = 0.03, *p* = 0.86).

### Corticosterone radioimmunoassay

Plasma CORT concentration was determined through radioimmunoassay (RIA) using extraction with diethyl ether (Fisher Chemical, Fair Lawn, New Jersey, USA) following the protocol described in Blas *et al.* (2005). All plasma samples were extracted in one extraction procedure and the extraction efficiency, estimated in three samples spiked with 5000 CPM of [^3^H]-CORT, was 95.5%. All reconstituted plasma extracts (300 μL of phosphate-buffered saline, 0.05 M and pH 7.6) were analysed in duplicate in four separate RIAs. Antiserum (C8784; lot 092M4784) and purified CORT (C2505, Lot 22K1439) for standards were obtained from Sigma-Aldrich Chemicals (Saint Louis, MO, USA). We measured assay variability as the coefficient of variation resulting from repeated measurement of six samples with a known CORT amount in each RIA. The intra- and inter-assay coefficients of variation were 5.7 and 11.6%, respectively. The average detection limit (ED 80 ± SD) was 22.49 ± 1.08 pg per assay tube, and CORT plasma values from all samples were above this limit.

### Statistical analyses

Differences in CORT among individuals across time (ca. 3, 15, 30, 45 and 60 min post-capture) were tested using linear mixed models in R 3.1.3, including habitat (urban or rural) and its interaction with time post-capture (linear and quadratic effects) as explanatory variables, and bird identity as a random term. Other factors may affect stress-induced CORT responses in birds, such as time of day (Romero and Remage-Healey, 2000), reproductive effort (Bonier *et al.*, 2011), body condition (Pérez-Rodríguez *et al.*, 2006) or sex (Blas *et al.*, 2011). Thus, we fitted into the model the sex of the individuals, their body condition (estimated as the residuals from a (log)body mass on (log)wing chord regression; female: *F*_1,600_ = 28.04, *p* < 0.001; male: *F*_1,409_ = 5.32, *p* = 0.022), the hour of the day and the brood size (as a proxy of reproductive effort) to control for their potentially confounding effects. The interaction between habitat and time post-capture was tested using Tukey *post hoc* tests for all pairwise comparisons among the 10 groups’ means (i.e. urban and rural, for ca. 3, 15, 30, 45 and 60 min post-capture).

We then assessed whether the CORT responses among individuals were similar in urban and rural areas or, conversely, whether they were more variable among rural than among urban ones, so that the latter represent a subset of the former. For this purpose, we compared a random intercept and a random slope model for each group of birds using log-likelihood tests. In random intercept models, the effect of the explanatory variable on the response is the same for all individuals (i.e. the CORT responses are similar for all individuals), while in random slope models their responses can differ (i.e. CORT responses are different among individuals).

**Figure 1:**
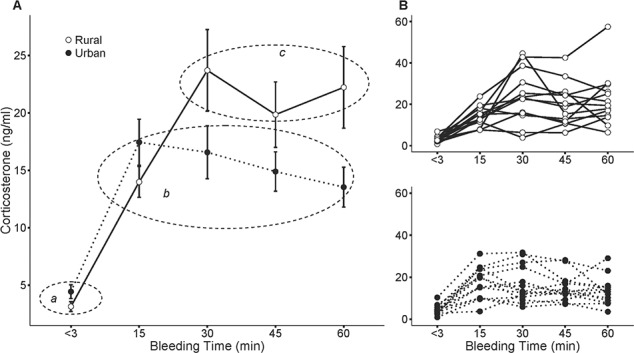
Average (panel **A**; mean ± standard error) and individual (panel **B**) plasma CORT levels at < 3 (baseline), 15, 30, 45 and 60 min post-capture in urban (black dots) and rural (white dots) breeding burrowing owls *Athene cunicularia*. Different letters indicate significant differences among groups of CORT values in the *post hoc* Tukey tests (dashed circles): *a* (i.e. baseline CORT levels of urban and rural owls), *b* (i.e. 15 to 60 min CORT levels of urban owls and 15 min CORT levels of rural owls) and *c* (i.e. 30 to 60 min CORT levels of rural owls).

Finally, we compared the maximum CORT titre in blood and the subsequent minimum levels reached between rural and urban birds to obtain a better visualization of CORT responses. For this purpose, we used linear models, controlling for body condition, brood size, sex and diel effects. Dependent variables in these models were (i) *absolute* maximum: the maximum stress-induced CORT level of each individual, (ii) *relative* maximum: the fold change from baseline to reach the maximum CORT level (calculated by dividing maximum by baseline CORT titre), (iii) *absolute* minimum post-peak: the minimum CORT level of each individual after reaching the maximum and (iv) *relative* minimum post-peak: the fold change from baseline to reach minimum post-peak CORT level (calculated dividing minimum post-peak by baseline CORT titre). When there was no reduction in CORT level after the maximum within our sampling timeframe, we considered the minimum post-peak values to be equal to the maximum ones.

## Results

Urban and rural birds showed statistically similar baseline CORT levels but differed in the time and magnitude of their stress-induced CORT responses. While urban individuals reached maximum CORT titres 15 min post-capture with a smooth decrease afterwards, rural individuals maintained a steady increase in plasma CORT up to 30 min after capture, when they reached a plateau (Fig. 1A, groups representing significant differences in the *post hoc* Tukey tests). These differences between urban and rural individuals remained statistically significant while controlling for sex, body condition, brood size and hour of the day (Table 1).

**Table 1:** Linear-mixed model explaining variability in CORT (baseline and stress-induced) levels in urban and rural breeding burrowing owls *Athene cunicularia* across the 60-min capture and restraint protocol.

Explanatory variables	Estimate	SE	*F*-test	*p*-value
Time	6.20	1.08	*F*_1,50_ = 31.23	<0.0001
Time^2^	−4.32	0.60	*F*_1,106_ = 52.02	<0.0001
Habitat (urban)	−2.71	2.21	*F*_1,47_ = 1.50	0.2263
Sex (females)	2.91	1.51	*F*_1,127_ = 3.72	0.0559
Body condition	−1.22	0.82	*F*_1,127_ = 2.17	0.1429
Hour of the day	0.84	0.88	*F*_1,127_ = 0.90	0.3450
Brood size	−0.41	0.80	*F*_1,127_ = 0.26	0.6078
Time^*^habitat	−4.03	1.49	*F*_1,50_ = 7.30	0.0094

Bird identity was included as a random term. SE: standard error.

**Table 2:** Linear models explaining variability in CORT levels in urban and rural breeding burrowing owls *Athene cunicularia.*

Dependent variable	Explanatory variables	Estimate	SE	*F*-test	*p*-value
*Absolute* maximum	Habitat (urban)	−4.02	4.63	*F*_1,21_ = 4.54	0.0450
	Body condition	−104.25	54.05	*F*_1,21_ = 5.12	0.0343
	Sex (females)	4.35	3.71	*F*_1,21_ = 1.78	0.1958
	Hour of the day	0.24	0.42	*F*_1,21_ = 0.25	0.6213
	Brood size	−1.78	1.43	*F*_1,21_ = 1.55	0.2266
*Relative* maximum	Habitat (urban)	−7.95	3.79	*F*_1,21_ = 4.72	0.0413
	Body condition	−1.69	44.21	*F*_1,21_ = 0.14	0.7145
	Sex (females)	1.47	3.03	*F*_1,21_ = 0.08	0.7832
	Hour of the day	0.37	0.34	*F*_1,21_ = 1.18	0.2898
	Brood size	−0.04	1.17	*F*_1,21_ = 0.01	0.9753
*Absolute* minimum post-peak	Habitat (urban)	−5.65	4.95	*F*_1,21_ = 4.78	0.0402
	Body condition	−94.35	57.75	*F*_1,21_ = 3.75	0.0664
	Sex (females)	2.26	3.96	*F*_1,21_ = 0.40	0.5354
	Hour of the day	0.23	0.44	*F*_1,21_ = 0.21	0.6498
	Brood size	−1.15	1.53	*F*_1,21_ = 0.57	0.4584
*Relative* minimum post-peak	Habitat (urban)	−7.50	3.59	*F*_1,21_ = 3.49	0.0759
	Body condition	15.47	41.88	*F*_1,21_ = 0.01	0.9700
	Sex (females)	0.66	2.87	*F*_1,21_ = 0.01	0.9780
	Hour of the day	0.42	0.32	*F*_1,21_ = 1.70	0.2069
	Brood size	−0.04	1.11	*F*_1,21_ = 0.01	0.9678

SE: standard error.

Models run separately for urban and rural birds showed that inter-individual CORT response were different for the two groups. While the random slope model did not fit data well for urban birds, showing that the CORT response was rather similar among individuals (likelihood ratio tests between the random intercept and the random slope models; *χ*^2^ = 0.29, df = 2, *p* = 0.8639), the response of rural birds was more variable, as shown by the better fit of the random slope model (likelihood ratio tests between the random intercept and the random slope models; *χ*^2^ = 13.99, df = 2, *p* = 0.0009). Despite these differences, the CORT response of both urban and rural birds followed a significant quadratic trend (Table S1). Also important, the more restricted range of CORT responses of urban individuals was encompassed within the wider variability displayed by their rural counterparts (Fig. 1B).

**Figure 2:**
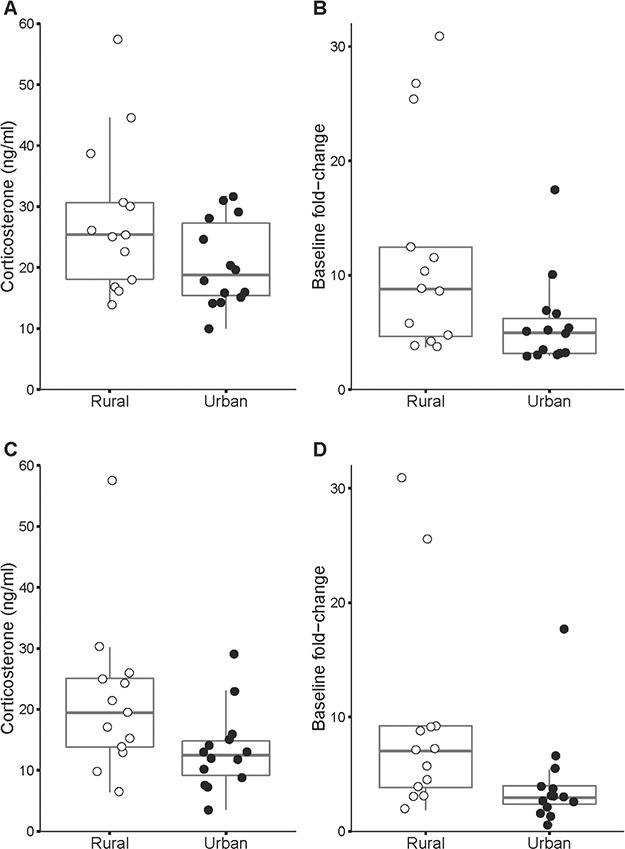
*Absolute* maximum (**A**), *relative* maximum (**B**), *absolute* minimum post-peak (**C**) and *relative* minimum post-peak (**D**) CORT levels in urban and rural breeding burrowing owls *Athene cunicularia*. Urban owls showed lower values compared to rural counterparts for all four measures (see Table 2 for results).

In accordance with the above results, *absolute* CORT maximum, *relative* CORT maximum, *absolute* CORT minimum post-peak and *relative* CORT minimum post-peak were also significantly higher in rural birds compared to urban owls, even while controlling for potential confounding effects such as sex, body condition, brood size and hour of the day (Table 2). Not only did rural birds show higher absolute CORT (*absolute* values, Fig. 2A and C), but they also reached more than twice the proportional CORT recorded by their urban counterparts (*relative* values, Fig. 2B and D). See also Fig. S1 for urban and rural comparison, after controlling for sex and body condition.

## Discussion

Our results show that, as predicted, urban and rural burrowing owls display similar baseline CORT levels but differ in their stress-induced responses. The adrenocortical response of urban owls reached its maximum 15 min after capture and started the termination sooner than rural birds, which reached a maximum later (30 min), reached higher plasma CORT levels and showed no sign of termination after 60 min. Also in agreement with our predictions, the HPA axis response was more homogeneous among urban birds, while rural owls showed a higher inter-individual variability that encompassed the range of values reported for urban owls. A lower stress-induced CORT response in urban than in non-urban individuals has been found in other bird species (Partecke *et al.*, 2006; Atwell *et al.*, 2012; Grunst *et al.*, 2014) and suggests that the close interaction with humans represents a source of stress, which can be tolerated only by particular phenotypes.

Previous studies performed in our burrowing owl populations have found that only individuals with less fear of humans (i.e. those with lower FID) can persist in the urban environment (Carrete and Tella, 2010, 2011). This results in an uneven occupation of territories by individuals with different tolerances to humans, which cannot be completely explained through habituation. Indeed, FID is highly repeatable throughout an individual’s adulthood (Carrete and Tella, 2010, 2013), and it is correlated with other behaviours such as exploration, anti-predator behaviour and dispersal within behavioural syndromes that differ between urban and rural birds (Carrete and Tella, 2017; Luna *et al.*, 2019a, 2019b). Recent full-genome sequencing approaches performed in different burrowing owl populations (including those of our study area) reinforce the idea that cities were colonized a few decades ago by a small number of founders from the surrounding natural areas (Mueller *et al.*, 2018), with an enrichment of different genes related to personality, behavioural control, memory and cognitive/learning functions (Mueller *et al.*, 2020). Heritability of FID has also been found to be high (Carrete *et al.*, 2016), although the lower values observed among urban birds can be related to differences in the ability of these individuals to adjust their FID adaptively (Vincze *et al.*, 2016). Thus, although much of our previous work points toward selective processes on behavioural traits during urban invasion by burrowing owls (i.e. through natural selection or matching habitat choice), phenotypic plasticity cannot be completely ruled out. Although less is known, it is likely that these same mechanisms that promote changes in the behavioural profiles of urban and rural populations also act to produce differences in their physiological profiles (i.e. the functioning of individual’s HPA axis) recorded within the frame of this study.

Bonier *et al*. (2007) suggested that the mechanism underlying the broader environmental tolerance of urban birds compared to rural conspecifics should involve physiological characteristics. Lowry *et al*. (2013) predicted that if bolder (i.e. less fearful) animals are more able to colonize urban environments, they should have a weaker stress-related CORT response compared to rural conspecifics to avoid the detrimental effects associated with chronic physiological stress typical of highly disturbed environments. Our results seem to support their prediction in the context of stress-related habitat matching. As we previously found when analysing feather CORT levels (Rebolo-Ifrán *et al.*, 2015), our current results show similar baseline CORT levels in urban and rural owls, likely reflecting their equal ability to afford daily and seasonal routines, as well as their similar overall energetic requirements (Romero *et al.*, 2009; Blas, 2015) or allostatic load (McEwen and Wingfield, 2003, 2010). Nevertheless, the significantly lower and less variable stress-induced CORT responses of urban owls in our study, which are embedded within the range of responses shown by rural conspecifics, suggest that the colonization of urban environments could be associated with particular physiological profiles able to cope with human disturbance. However, our results do not allow us to know whether these differences in the physiological responses of urban and rural individuals are plastic responses toward differences in the degree of human perturbation or result from a non-random distribution of individuals showing fixed, unchanging, physiological profiles. Although some studies on this topic have been conducted (Schoenemann and Bonier, 2018; Taff *et al.*, 2018), there is no agreement regarding how much of the stress-induced response of individuals remains constant throughout their lives and how much corresponds to individual responsiveness to environmental variation. This information is paramount to understanding the mechanisms underlying the differences observed between urban and rural individuals, as natural selection and matching habitat choice rely on the assumption that the traits of interest remain constant through an individual’s lifespan and are heritable, while habituation (phenotypic plasticity) assumes that organisms are able to respond to variation in the environment by modifying their phenotype (although there are studies showing that plasticity *per se* can also be heritable and genetically fixed; Hallsson and Björklund, 2012; Scheiner *et al.*, 2012; Gomez-Mestre and Jovani, 2013). Partecke *et al.* (2006), for instance, have found lower stress-induced CORT responses in urban than in rural blackbirds (*Turdus merula*) reared in a common-garden experiment, suggesting a genetically based difference. Similar results were obtained by Atwell *et al.* (2012) using dark-eyed juncos (*Junco hyemalis*), without ruling out early developmental effects. CORT responses have also been found to be heritable in some bird species (Evans *et al.*, 2006; Angelier *et al.*, 2011; Rensel and Schoech, 2011; Cox *et al.*, 2016), including another owl species (*Tyto alba*) where baseline and stress-induced CORT levels are genetically correlated and heritable (Béziers *et al.*, 2019). However, other studies have shown a low individual consistency in adrenocortical responses (Romero and Reed, 2008; Ouyang *et al.*, 2011; Lendvai *et al.*, 2015) and CORT levels as defined by genetic components and environmental effects (Jenkins *et al.*, 2014; Ouyang *et al.*, 2019), thus complicating our understanding of the mechanisms underlying the observed patterns.

An important point that should be addressed to understand the role of phenotypic plasticity, natural selection or matching habitat choice in the observed differences between urban and rural birds is the link between CORT response and fitness. Although it is accepted that high levels of CORT secretion may result in chronic physiological stress with detrimental effects (Raber, 1998; Sapolsky *et al.*, 2000; Romero, 2004), the relationships between CORT levels and fitness components in animals are not fully understood (Bonier *et al.*, 2009; Crespi *et al.*, 2013). Nonetheless, different measurements of CORT levels have been significantly related to subsequent survival in some bird species (Brown *et al.*, 2005; Blas *et al.*, 2007; Goutte *et al.*, 2010; Koren *et al.*, 2012; Harms *et al.*, 2015), including our study species (Rebolo-Ifrán *et al.*, 2015). There, we found a quadratic relationship between feather CORT levels and survival of urban burrowing owls, indicating stabilizing rather than directional selection favouring intermediate CORT levels. The absence of such a relationship in rural owls was attributed to a higher predation pressure in this habitat (Rebolo-Ifrán *et al.*, 2017), which could mask any CORT-survival relationship (Rebolo-Ifrán *et al.*, 2015).

Understanding the relative importance of phenotypic plasticity, natural selection and habitat matching choice to explain differences in the CORT responses of urban and rural birds would undoubtedly fill large gaps, not only in our knowledge of stress responses but also in terms of biodiversity conservation. Urbanization is currently one of the faster, longer-lasting sources of habitat transformation worldwide (Vitousek *et al.*, 1997) and is already eroding and homogenizing biodiversity (McKinney, 2008; Aronson *et al.*, 2014; Sol *et al.*, 2014), also reducing phylogenetic diversity (Sol *et al.*, 2017). Moreover, impacts on biodiversity conservation are expected to increase in the near future, as by 2050 it is expected that almost 70% of the world human population will be living in cities (United Nations, 2016). However, some species such as our study model can thrive better in cities than in their non-urban surrounding habitats (Rebolo-Ifrán *et al.*, 2017), and some cities even constitute conservation hotspots for threatened species (Ives *et al.*, 2016; Luna *et al.*, 2018), which could serve as population and genetic stocks for conservation programs (Gibson and Yong, 2017). Therefore, there is an increasing motivation to make urban environments friendly for wildlife (Miller and Hobbs, 2002; Dearborn and Kark, 2010) and to predict which species will be able to cope with urbanization.

The ability of species to thrive (or not) in urban environments has been associated with behavioural (Carrete and Tella, 2011; Sol *et al.*, 2014), ecological (Silva *et al.*, 2016) and biogeographic traits (González-Oreja, 2011). However, these comparative analyses fail to explain a large component of the variance, which may be attributed to random processes and/or to the role played by other overlooked mechanisms. The identification of the mechanisms causing the physiological differences in stress responses observed between individuals living in urban and non-urban environments may be paramount to a better understanding of these processes and to forecast further changes in biodiversity and species population dynamics. For example, if between-individual differences in the stress-response of some species mainly arise from phenotypic plasticity, we may expect that any individual of these highly plastic species would be able to physiologically cope with urbanization. These species could easily colonize urban habitats and increase their local and even global population sizes by exploiting these new habitats, provided they offer adequate food (Geis and Pomeroy, 1993; Robb *et al.*, 2008) and resources for reproduction and survival (Chamberlain *et al.*, 2009; Deviche and Davies, 2014), and relief from predation pressure typical in urban environments (Díaz *et al.*, 2013; Rebolo-Ifrán *et al.*, 2017; Luna *et al.*, 2018). However, if differences between urban and rural individuals are related to differences in the colonization of these habitats by individuals showing different, consistent adrenocortical responses, only a proportion of the population (i.e. those individuals pre-adapted to tolerate high human disturbance) could colonize the cities. In terms of invasion biology (Kolar and Lodge, 2001), smaller propagule sizes would slow down a colonization process, highlighting the need to preserve rural populations even among those taxa defined as urban exploiters. Moreover, the effects on population size would greatly differ depending on whether the colonization occurs through natural selection or habitat matching choice. In the first case, urban colonization would be a random process where only pre-adapted individuals would survive, thus reducing the population size of the species. In the second case, however, pre-adapted individuals could actively colonize the city and thrive well there, without detrimental fitness consequences.

Although considerable advances have been made in the field of physiological ecology, further research is still needed. Hypothesis-driven research is particularly important, and future work should be mainly focused on disentangling the mechanisms allowing wildlife adaptation to urban areas. Understanding the relative role played by each of these mechanisms in a wide range of species would allow us to properly design conservation strategies in an increasingly urbanized world.

## Authors’ contributions

A.P., M.C., J.B. and J.L.T. conceived the study. A.P. conducted the fieldwork. S.C. and T.A.M. performed the laboratory analyses. A.P., M.C. and J.B. analysed the data. A.P., J.L.T., J.B. and M.C. wrote the paper, and S.C. improved it with suggestions. All authors agree to be held accountable for the content therein and approve the final version of the manuscript.

## Funding

This work was supported by Fundación Repsol, projects CGL2012-31888 and CGL2015-71378 (MEC, Spain) and COOPA20049 (CSIC, Spain). A.P. was supported by International Fellowships Programme Caixa-Severo Ochoa.

## Supplementary Material

Fig_S1_residuals_coaa054

Palma_et.al_ConPhy_spm_coaa054
