# Supplementary figures and images for: Differences in adrenocortical responses between urban and rural burrowing owls: poorly-known underlying mechanisms and their implications for conservation
Source: Conserv Physiol. 2020 Jul 6;8(1):coaa054. doi: 10.1093/conphys/coaa054 (PMC7336563; doi:10.1093/conphys/coaa054)

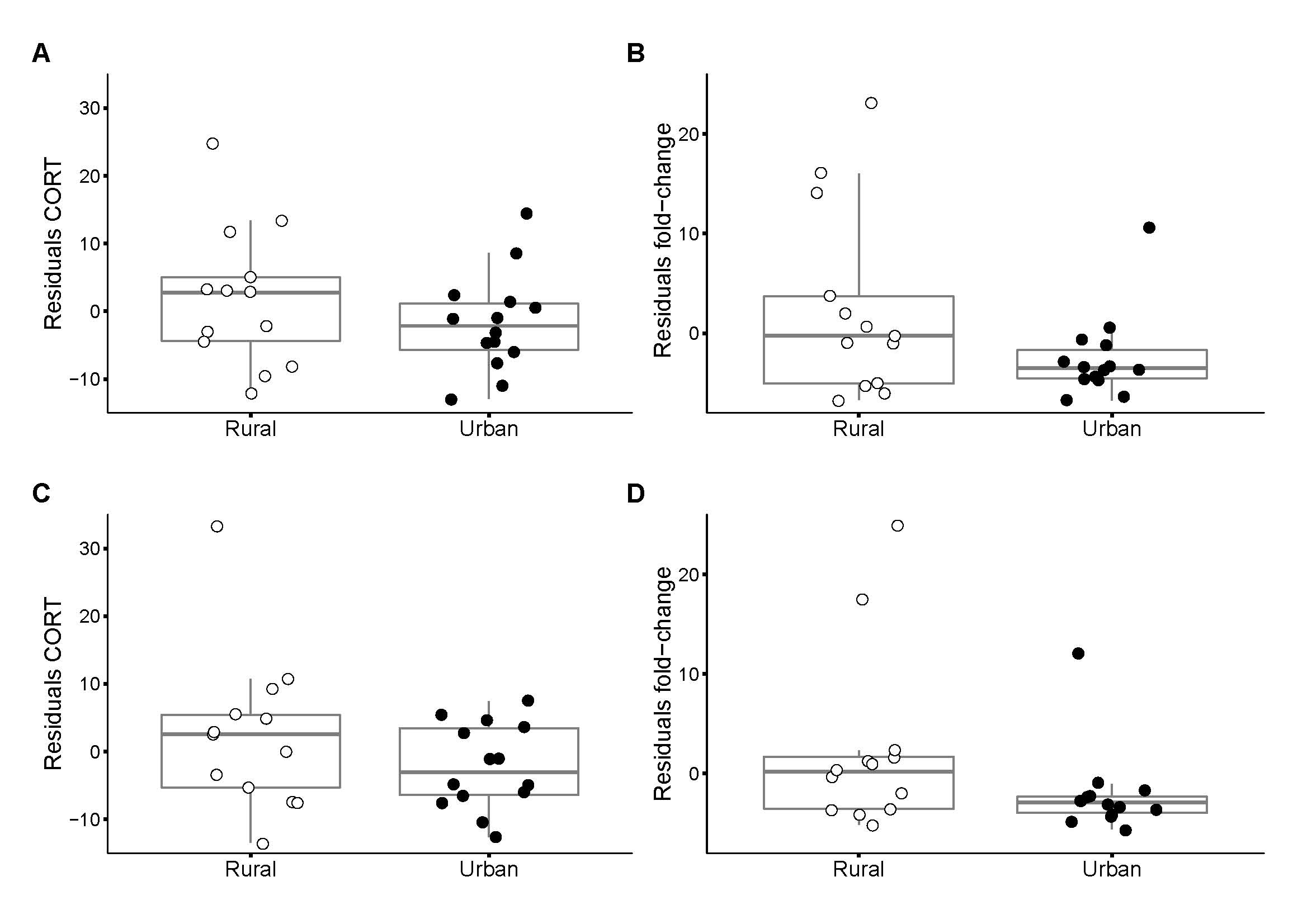

Supplement: Fig_S1_residuals_coaa054 [file fig_s1_residuals_coaa054.jpeg]
