# Supplementary material for: Differences in adrenocortical responses between urban and rural burrowing owls: poorly-known underlying mechanisms and their implications for conservation
Source: Conserv Physiol. 2020 Jul 6;8(1):coaa054. doi: 10.1093/conphys/coaa054 (PMC7336563; doi:10.1093/conphys/coaa054)

# Supplementary material

# Table and legends figure

**Table S1.** Linear-mixed models explaining variability in CORT (baseline and stress-induced) levels in urban and rural breeding burrowing owls *Athene cunicularia* across the 60-minute capture and restraint protocol. SE: standard error. ID: individual identity.

**Fig. S1.** Residual CORT levels plots from the regression between CORT levels and body condition and sex for *absolute* maximum (A), *relative* maximum CORT (B), *absolute* minimum post-peak (C), and *relative* minimum post-peak (D) CORT levels in urban and rural breeding burrowing owls *Athene cunicularia*.

**Table S1.** Linear-mixed models explaining variability in CORT (baseline and stress-induced) levels in urban and rural breeding burrowing owls *Athene cunicularia* across the 60-minute capture and restraint protocol. SE: standard error. ID: individual identity.

| Explanatory variables | Urban | | | |  | Rural | | | |
| --- | --- | --- | --- | --- | --- | --- | --- | --- | --- |
|  | Estimate | SE | F-test | p-value |  | Estimate | SE | F-test | p-value |
| Time | 2.24 | 0.62 | F_1,58_ = 13.07 | 0.0006 |  | 5.91 | 1.32 | F_1,14_ = 20.15 | 0.0005 |
| Time^2^ | -4.25 | 0.74 | F_1,56_ = 33.09 | <0.0001 |  | -4.41 | 0.90 | F_1,51_ = 24.23 | <0.0001 |
| Sex (females) | 3.55 | 2.27 | F_1,14_ = 2.43 | 0.1412 |  | 4.48 | 2.74 | F_1,29_ = 2.68 | 0.1122 |
| Body condition | -1.96 | 1.03 | F_1,14_ = 3.6 | 0.0786 |  | -1.37 | 1.80 | F_1,29_ = 0.58 | 0.4536 |
| Hour of the day | -0.30 | 1.11 | F_1,14_ = 0.07 | 0.7907 |  | 5.13 | 1.82 | F_1,29_ = 7.98 | 0.0085 |
| Brood size | 0.34 | 1.15 | F_1,14_ = 0.09 | 0.7698 |  | -1.28 | 1.47 | F_1,29_ = 0.76 | 0.3896 |
|  | Random term: (Time + Time^2^)*ID | | | |  | Random term: (Time + Time^2^)*ID | | | |

**Fig. S1.** Residual CORT levels plots from the regression between CORT levels and body condition and sex for *absolute* maximum (A), *relative* maximum CORT (B), *absolute* minimum post-peak (C), and *relative* minimum post-peak (D) CORT levels in urban and rural breeding burrowing owls *Athene cunicularia*.


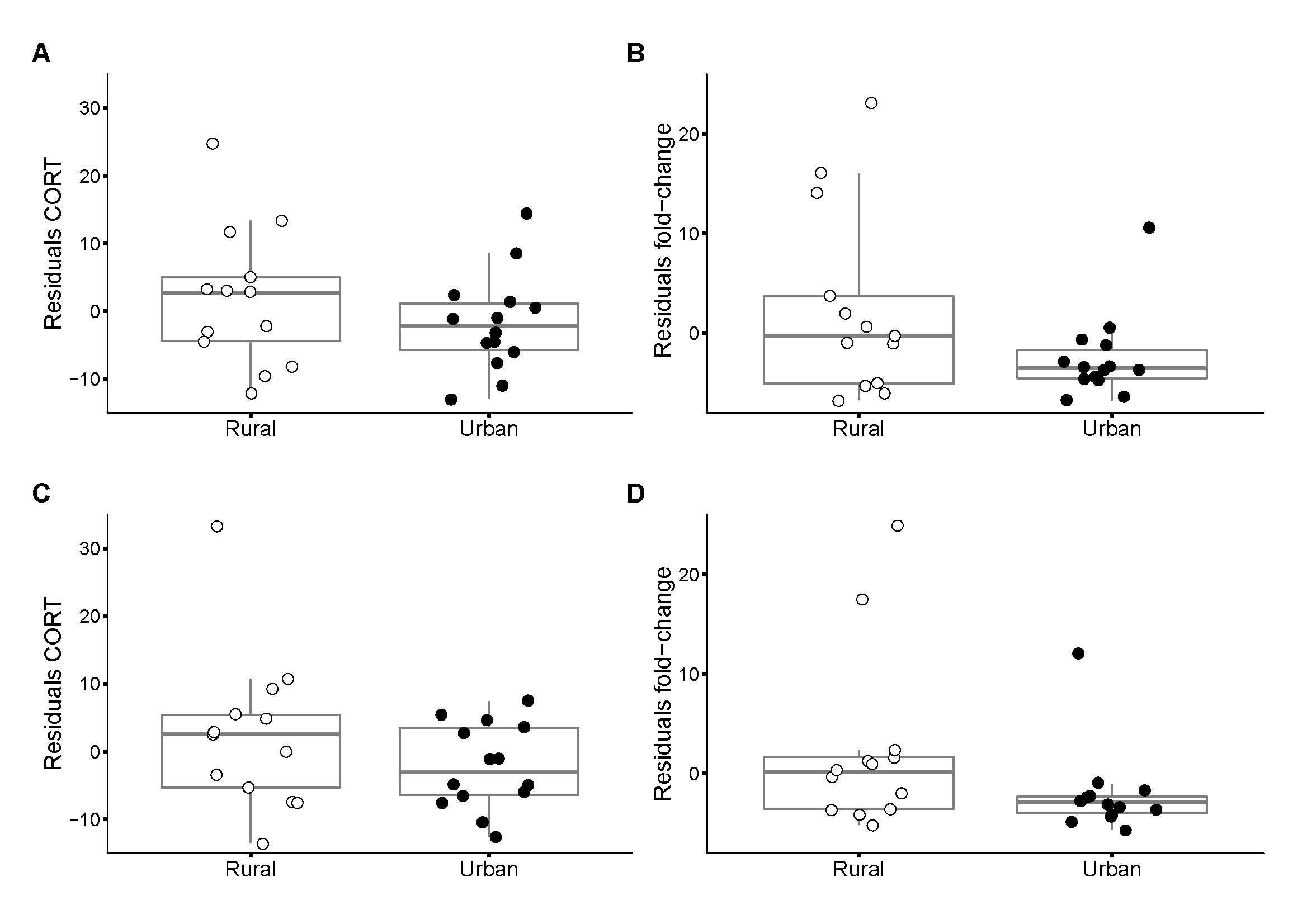

Supplement: Palma_et.al_ConPhy_spm_coaa054 [file palma_et.al_conphy_spm_coaa054.doc]
